# Effective reduction of cadmium accumulation in rice grain by expressing *OsHMA3* under the control of the *OsHMA2* promoter

**DOI:** 10.1093/jxb/ery107

**Published:** 2018-03-17

**Authors:** Ji Feng Shao, Jixing Xia, Naoki Yamaji, Ren Fang Shen, Jian Feng Ma

**Affiliations:** 1 Institute of Plant Science and Resources, Okayama University, Chuo, Kurashiki, Japan; 2 State Key Laboratory of Soil and Sustainable Agriculture, Institute of Soil Science, Chinese Academy of Sciences, Nanjing, China; 3 State Key Laboratory of Conservation and Utilization of Subtropical Agro-bioresources, College of Life Science and Technology, Guangxi University, Nanning, China

**Keywords:** Cadmium, node, OsHMA2, OsHMA3, transgenic rice, vacuolar sequestration

## Abstract

Reducing cadmium (Cd) accumulation in rice grain is an important issue for human health. The aim of this study was to manipulate both expression and tissue localization of OsHMA3, a tonoplast-localized Cd transporter, in the roots by expressing it under the control of the *OsHMA2* promoter, which shows high expression in different organs including roots, nodes, and shoots. In two independent transgenic lines, the expression of *OsHMA3* was significantly enhanced in all organs compared with non-transgenic rice. Furthermore, OsHMA3 protein was detected in the root pericycle cells and phloem region of both the diffuse vascular bundle and the enlarged vascular bundle of the nodes. At the vegetative stage, the Cd concentration in the shoots and xylem sap of the transgenic rice was significantly decreased, but that of the whole roots and root cell sap was increased. At the reproductive stage, the concentration of Cd, but not other essential metals, in the brown rice of transgenic lines was decreased to less than one-tenth that of the non-transgenic rice. These results indicate that expression of *OsHMA3* under the control of the *OsHMA2* promoter can effectively reduce Cd accumulation in rice grain through sequestering more Cd into the vacuoles of various tissues.

## Teaser

Cd accumulation in rice grain was effectively reduced by expressing *OsHMA3*, which encodes a transporter responsible for vacuolar Cd sequestration, under the control of the *OsHMA2* promoter.

## Introduction

Cadmium (Cd) is a class 1 carcinogen that can be accumulated in the human body over time from ingestion of Cd-containing food (Clemens and Ma, 2016). Excessive intake may result in damage to kidney tubules, rhinitis, emphysema, as well as other chronic disorders (McLaughlin *et al.*, 1999). Therefore, Cd intake through the food chain poses a serious threat to human health and food security (Clemens and Ma, 2016). Rice is an important staple food and a major source of Cd intake (Watanabe *et al.*, 2004; Cheng *et al.*, 2006). ‘Itai-itai disease’ is the result of intake of Cd-containing rice. The Codex Alimentarius Commission/World Health Organization with responsibility for the safety of food and human health has set 0.4 mg Cd kg ^−1^ in polished rice grain as the maximum permissible limit (Codex Alimentarius Commission of Food and Agriculture Organization, 2006), but rice produced in some areas often exceeds this. Therefore, it is a very important issue for human health to limit transfer of Cd from the soil to the grain in rice.

There are several steps for Cd transfer from soil to grain including at least uptake by the roots, root-to-shoot translocation, and distribution to the grain (Clemens and Ma, 2016). Several transporters involved in these processes have been identified in rice. The uptake of Cd is mediated by OsNramp5, a member of the natural resistance-associated macrophage protein family (Ishimaru *et al.*, 2012; Sasaki *et al.*, 2012). It is polarly localized at the distal side of both root exodermis and root endodermis (Sasaki *et al.*, 2012). The expression of *OsNramp5* is not induced by Cd. Knockout of *OsNramp5* resulted in complete loss of Cd uptake, indicating that OsNramp5 is a major transporter for Cd uptake and is responsible for transporting Cd from soil solution to rice root cells. After the uptake, part of the Cd is subsequently sequestered into the vacuoles by OsHMA3, a P-type heavy metal ATPase (Ueno *et al.*, 2010; Miyadate *et al.*, 2011). OsHMA3 is localized to the tonoplast of all root cells (Ueno *et al.*, 2010). The expression of *OsHMA3* is also not induced by Cd. Loss of function of this gene resulted in high Cd accumulation in the shoots and grains (Ueno *et al.*, 2010). On the other hand, the remaining part of the Cd taken up by OsNramp5 is translocated to the shoots, which is mediated by OsHMA2, a homolog of OsHMA3 (Satoh-Nagasawa *et al.*, 2012; Takahashi *et al.*, 2012; Yamaji *et al.*, 2013). However, differing from OsHMA3, OsHMA2 is a plasma membrane-localized transporter for Cd and is localized at the pericycle of the roots (Yamaji *et al.*, 2013). *OsHMA2* is constitutively expressed in the roots at the vegetative stage, and knockout of this gene resulted in lower root-to-shoot translocation of Cd (Satoh-Nagasawa *et al.*, 2012; Takahashi *et al.*, 2012; Yamaji *et al.*, 2013).

On the other hand, at the reproductive stage, two transporters (OsHMA2 and OsLCT1) responsible for the distribution of Cd to the grains have been identified. In addition to the roots, *OsHMA2* is also expressed in the nodes (Yamaji *et al.*, 2013). Especially, it is highly expressed in node I, the uppermost node at the reproductive stage. In nodes, OsHMA2 is localized at the phloem of enlarged vascular bundles and diffuse vascular bundles (Yamaji *et al.*, 2013). Knockout of *OsHMA2* resulted in decreased concentration of Cd in the upper nodes and reproductive organs compared with wild-type rice. Therefore, OsHMA2 is involved in reloading Cd from the intervening parenchyma tissues into the phloem of diffuse vascular bundles (Yamaji *et al.*, 2013). On the other hand, OsLCT1 (*Oryza sativa* low-affinity cation transporter 1) seems to be also involved in the intervascular transfer of Cd (Uraguchi *et al.*, 2011). OsLCT1 is localized to the plasma membrane and shows efflux transport activity not only for Cd but also for K, Mg, Ca and Mn (Uraguchi *et al.*, 2011). *OsLCT1* is expressed around the enlarged vascular bundles and diffuse vascular bundles in the node at the reproductive stage (Uraguchi *et al.*, 2011). Knockdown of *OsLCT1* resulted in decreased Cd concentration in phloem and grains (Uraguchi *et al.*, 2011). However, in contrast to *OsHMA2*, which is highly expressed in the nodes throughout reproductive growth (Yamaji *et al.*, 2013), expression of *OsLCT1* in the nodes was only observed at the ripening stage (Uraguchi *et al.*, 2011). OsLCT1 might be involved in efflux of Cd from the phloem within the nodes at this stage.

Several attempts have been made to reduce Cd accumulation in the rice grain by manipulating the transporter genes described above. However, although Cd accumulation was reduced in the grain of the genetically modified rice, a penalty of reduced growth and yield was observed in some cases. This is because some transporters identified for Cd are also involved in transporting essential metals (Clemens and Ma, 2016). For example, OsNramp5 is also a major transporter for Mn (Sasaki *et al.*, 2012). Therefore, knockout of its gene also reduces Mn uptake, resulting in decreased rice yield especially under Mn-limited condition (Sasaki *et al.*, 2012), although contradictory results were also reported due to different growth conditions (Ishikawa *et al.*, 2012; Tang *et al.*, 2017). Knockout of *OsHMA2* also resulted in decreased rice yield because it is also required for Zn transport (Yamaji *et al.*, 2013). An exception is overexpression of *OsHMA3* in rice. The expression of *OsHMA3* is very low in native rice cultivars (Ueno *et al.*, 2010). Overexpression of *OsHMA3* selectively reduced accumulation in the grain of Cd but not of Zn and Fe (Ueno *et al.*, 2010; Sasaki *et al.*, 2014). Although OsHMA3 also transports Zn, Zn homeostasis in the shoots seems to be maintained by up-regulating five genes related to Zn transport (Sasaki *et al.*, 2014).

The aim of this study was to find a way to efficiently reduce Cd accumulation in rice grains by utilizing different expression pattern and properties of two Cd transporter genes, *OsHMA3* and *OsHMA2*. *OsHMA3* is mainly expressed in the roots and is involved in vacuolar sequestration of Cd (Ueno *et al.*, 2010), while *OsHMA2* is expressed in the roots and nodes (Yamaji *et al.*, 2013). Therefore, if *OsHMA3* is expressed under the control of the *OsHMA2* promoter, there is a possibility that Cd will be sequestered into the vacuoles at the roots and nodes before loading to the grain, resulting in decreased Cd accumulation in rice grain. Our results support this possibility, and this provides an effective way to reduce Cd accumulation in the grains of rice grown in Cd-contaminated soils.

## Materials and methods

### Generation of transgenic plants

To construct the *ProOsHMA2-OsHMA3* DNA fusion, the promoter of *OsHMA2* (2.13 kb upstream of the translational start) was amplified by PCR from cv. Nipponbare genomic DNA using primers 5′-*AAGCTT*CAACTCTTTTCTTCCGTTTGTGT-3′ (*Hin*dIII site italicized) and 5′-*GGATCC*CTCTCCTCACTCTCTCCCTCTT-3′ (*Bam*HI site italicized). Using *Hin*dIII and *Bam*HI, the amplified fragment was cloned into pPZP2H-lac carrying the terminator of the nopaline synthase gene, producing the *OsHMA2* promoter construct. The *OsHMA3* cDNA containing a *Bam*HI restriction site was amplified from Nipponbare by RT-PCR using the primers 5′-A*ggatcc*ATGGCCGGAAAGGATGAGG-3′ (*Bam*HI site italicized) and 5′-T*ggatcc*GCAACATCATCCTTTCACTTCACC-3′ (*Bam*HI site italicized). Using *Bam*HI, the amplified fragment was cloned into pPZP2H-lac carrying the *OsHMA2* promoter and the terminator of the nopaline synthase gene, resulting in the *OsHMA2* promoter-*OsHMA3* cDNA construct (*ProOsHMA2-OsHMA3*) (see Supplementary Fig. S1 at *JXB* online). This construct was transformed into *Agrobacterium tumefaciens* (strain EHA101), followed by transforming to calluses derived from Nipponbare according to Hiei *et al.* (1994).

### RNA isolation and gene expression analysis

To investigate the expression level of *OsHMA3* in the transgenic lines, roots, shoot basal region (0.5 cm from the root-to-shoot junction), and shoots of 28-day-old seedlings grown hydroponically were separately sampled. The total RNA was extracted with an RNeasy Plant Mini Kit (Qiagen). One microgram of total RNA was used for first strand cDNA synthesis using a SuperScript II kit (Invitrogen), following the manufacturer’s instructions with an oligo (dT)20 primer. The expression was determined with SYBR Premix Ex Taq^TM^ (Takara) by Mastercycler ep realplex (Eppendorf). The primer sequences for quantitative RT-PCR were 5′-CATAGTGAAGCTGCCTGAGATC-3′ and 5′-GATCAAACGCATAGCAGCATCG-3′ for *OsHMA2*, 5′-TCCATCCAACCAAACCCGGAAA-3′ and 5′-TGCCAATGTCCTTCTGTTCCCA-3′ for *OsHMA3*, 5′-CGTCATGGCTGTCGTCATGATCTG-3′ and 5′-AATGGGGTGATAGAAATCGAACATG-3′ for *ZIP1*, 5′-GCATTGTTCAGGCTAATTTTAAGG-3′ and 5′-GGCAGTTGAGCTATGCACATTG-3′ for *ZIP3*, 5′-TCACTGAGGCCGTCGTCAATCAGG-3′ and 5′-ACGACAAGTGCGGTCGAGCTGT-3′ for *ZIP4*, 5′-GGTGCAGAGCAAAGGCAAGCT-3′ and 5′-AATTTCCTCTACATTAGTCCCTGA 3′ for *ZIP8*, 5′-ATCTTCTTCTCGCTAACCACAC-3′ and 5′-GCAGCCGCTGCGTCGAGAAT-3′ for *ZIP9*, and 5′-GCTCAGTTAAAGAACTTCTCTGC-3′ and 5′-CGACATCGAGTCCAGAATTCC-3′ for *ZIP10*. *Histone H3* was used as an internal standard with the primers 5′-GGTCAACTTGTTGATTCCCCTCT-3′ and 5′-AACCGCAAAATCCAAAGAACG-3′. The relative expression was normalized by the ΔΔ*C*_t_ method using CFX Manager software (Bio-Rad). The expression pattern of *OsHMA2* and *OsHMA3* in different organs was compared with semi-quantitative PCR.

### Immunostaining of OsHMA3 in transgenic rice

To investigate the localization of OsHMA3 in the transgenic line, we performed immunostaining with an antibody against OsHMA3 used previously (Ueno *et al.*, 2010). The root segments, shoot basal region, and node I were sampled for immunostaining. The procedures for immunostaining were the same as described before (Yamaji and Ma, 2007). Fluorescence of secondary antibody (Alexa Fluor 555 goat anti-rabbit IgG; Molecular Probes) was observed with a confocal laser scanning microscope (LSM700; Carl Zeiss).

### Phenotypic analysis of transgenic plants at vegetative stage

Seeds of two transgenic lines (B9 and B17) and non-transgenic rice (cv. Nipponbare) were soaked in water for 2 d at 30 °C in the dark and the geminated seeds were transferred to nylon nets floating on a solution containing 0.5 mM CaCl_2_ in a 1.2 liter pot and grown for another 5 d. The seedlings were then transferred to a 3.5 liter pot containing 1/2 Kimura B nutrient solution (pH 5.6). The solution contained the following macronutrients (mM): MgSO_4_ (0.28), (NH_4_)_2_SO_4_ (0.18), Ca(NO_3_)_2_ (0.18), KNO_3_ (0.09), and KH_2_PO_4_ (0.09); and micronutrients (µM): Fe-EDTA (20), H_3_BO_3_ (3), MnCl_2_ (0.5), CuSO_4_ (0.2), ZnSO_4_ (0.4), and (NH4)_6_Mo_7_O_24_ (1). The solution was renewed every 2 d.

To compare Cd accumulation in different organs, seedlings of transgenic and non-transgenic lines (17 d old) were exposed to a nutrient solution containing 0, 0.1, and 1 µM Cd as CdSO_4_. The solution was changed every 2 d. The plants were grown in a controlled glasshouse at 25–30 °C under natural light. After 8 d, the roots were washed with 5 mM cold CaCl_2_ three times and separated from the shoots. The shoot basal region (0.5 cm from the root-to-shoot junction) was excised by razor from the shoots. All samples were subjected to determination of mineral elements as described below. Three biological replicates were made for each treatment in different pots.

### Collection of xylem sap and root cell sap

For collection of xylem sap, seedlings (17 d old) were exposed to a nutrient solution containing 1 µM Cd. The solution was changed every 2 d. After 8 d, the shoot (2 cm above the root) was excised with a razor, and the xylem sap was collected with a micropipette for 45 min after decapitation of the shoot.

For collection of root cell sap, the roots of the seedlings after Cd exposure as described above were washed with 5 mM cold CaCl_2_ three times and blotted with a paper towel, followed by placing on a filter in a tube. The samples were frozen at −80 °C till use. Before collection of root cell sap, the sample was thawed at room temperature, followed by centrifugation at 20 600 *g* for 10 min. The concentration of Cd and other metals in the xylem sap and root cell sap was determined as described below.

### Analysis of Cd accumulation in grain of transgenic rice

To investigate Cd accumulation in rice grain, two transgenic lines (B9 and B17) and a non-transgenic line were grown in a Cd-contaminated soil until ripening. Seedlings (28 d old) prepared hydroponically as described above were transplanted to a pot containing 3.5 kg soil supplemented with 1.0 mg kg^−1^ Cd as CdSO_4_ with three replicates. The plants were grown in a controlled glasshouse at 25–30 °C under natural light from 19 July to 7 November 2012. The plants were grown under submerged conditions until flowering time and then changed to upland conditions by watering with tap water every day. At harvest, the plants were separated into brown rice, node I, flag leaf, and the remaining part of straw. The concentration of Cd in these organs was determined with inductively coupled plasma mass spectrometry (ICP-MS) after digestion as described below.

### Determination of Cd and other metals

All samples were dried at 70 °C in an oven for at least 3 d. The dried samples were digested with concentrated HNO_3_ (60%) at a temperature up to 140 °C. The metal concentration in the digested solution, xylem sap, and root cell sap was determined by ICP-MS (7700X; Agilent Technologies) after appropriate dilution.

### Statistical analysis

The analysis of significance was performed by one-way ANOVA, followed by Tukey’s test. Significance of differences at *P*<0.05 is shown by different letters in the figures.

## Results

### Expression of *OsHMA3* in transgenic lines

**Fig. 1.**
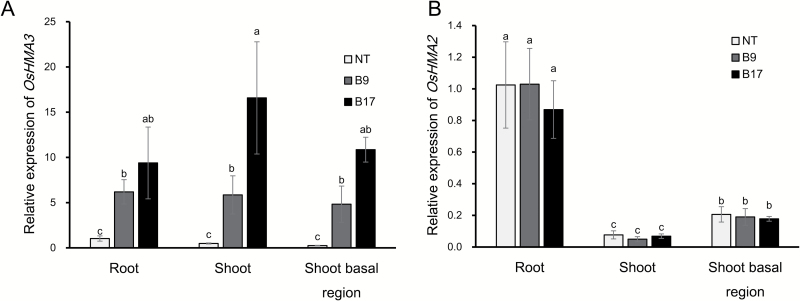
Expression of *OsHMA3* in different organs of transgenic and non-transgenic lines. Different organ samples were taken from seedlings (28 d old) of two independent transgenic lines carrying *OsHMA3* under the control of *OsHMA2* and a non-transgenic (NT) line. The expression of *OsHMA3* (A) and *OsHMA2* (B) was determined by quantitative RT-PCR. *HistoneH3* was used as an internal standard. Expression relative to the roots of the non-transgenic line is shown. Data represent means ±SD (*n=*3). Statistical comparison was performed by one-way ANOVA, followed by Tukey’s multiple comparison test. Different lower-case letters indicate significant difference at *P*<0.05.

We obtained two independent transgenic lines (B9 and B17) carrying *OsHMA3* under the control of the *OsHMA2* promoter (see Supplementary Fig. S1). Expression analysis showed that the expression level of *OsHMA3* was significantly increased in the roots, shoot basal region including basal nodes, and shoots of two transgenic lines compared with non-transgenic line (Fig. 1A). Among the transgenic lines, B17 showed a higher *OsHMA3* expression than B9. By contrast, the expression of *OsHMA2* did not differ between transgenic and non-transgenic lines (Fig. 1B), indicating that introduction of *OsHMA2* promoter only enhanced the expression of *OsHMA3*. These expression patterns were similar to their native ones; *OsHMA2* was highly expressed in the roots, shoots, and shoot basal region, while *OsHMA3* was mainly expressed in the roots at a low level (Supplementary Fig. S2).

### Tissue localization of OsHMA3 in transgenic line

**Fig. 2.**
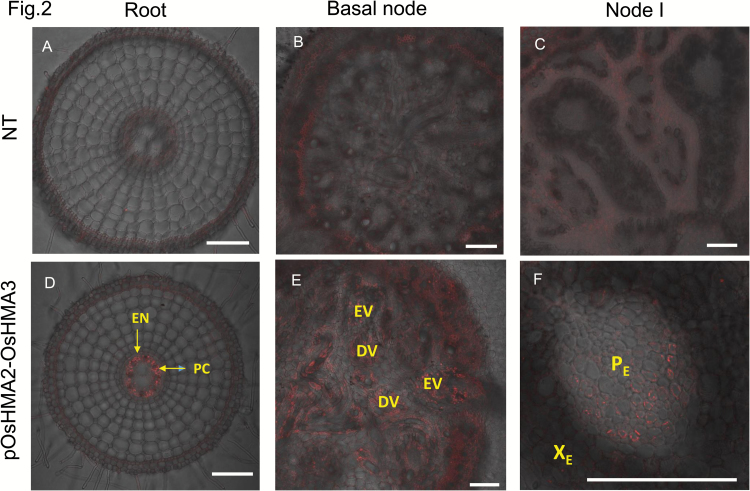
Localization of OsHMA3 in transgenic and non-transgenic lines. Immunostaining with an OsHMA3 antibody was performed in root (A, D), basal node (B, E), and node I (C, F) of non-transgenic line (A, B, C) and transgenic line carrying *OsHMA3* under the control of *OsHMA2* promoter (D, E, F). Red indicates OsHMA3-speciﬁc signal. Scale bar is 100 µm. DV, diffuse vascular bundle; EN, endodermis; EV, enlarged vascular bundle; PC, pericycle; P_E_, phloem of enlarged vascular bundle; X_E_, xylem of enlarged vascular bundle.

To investigate whether OsHMA3 localization was altered under the control of the *OsHMA2* promoter, we performed immunostaining using an antibody specific for OsHMA3 (Ueno *et al.*, 2010). To make all samples comparable, we observed all cross sections under the same conditions. In the non-transgenic line, signal was hardly detected in all tissues due to the detection condition used (Fig. 2A–C). By contrast, a strong signal was detected in the root pericycle and phloem region of both the diffuse vascular bundle and the enlarged vascular bundle of basal nodes of the transgenic line (Fig. 2D–E). In node I of the transgenic line, a strong signal was also detected in the phloem region of the diffuse vascular bundle and the enlarged vascular bundle (Fig. 2F). These results indicate that introduction of the *OsHMA2* promoter altered the tissue localization of OsHMA3.

### Phenotypic analysis of transgenic lines at vegetative growth stage

**Fig. 3.**
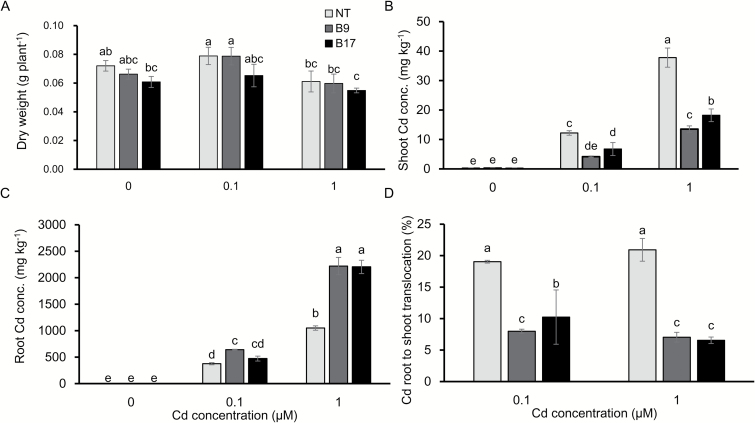
Comparison of biomass and Cd accumulation between transgenic and non-transgenic lines. (A) Plant dry weight. (B, C) Concentration of Cd in the shoots (B) and roots (C). (D) Root-to-shoot translocation rate of Cd. Seedlings (17 d old) of transgenic lines carrying *OsHMA3* under the control of *OsHMA2* promoter were exposed to different Cd concentrations (0, 0.1, 1 µM) in a 1/2 Kimura B nutrient solution for 8 d. The Cd concentration in the roots and shoots was determined by ICP-MS. Root-to-shoot translocation rate was calculated by Cd content in shoots/total Cd content×100. Data are means ±SD (*n=*3). Statistical comparison was performed by one-way ANOVA, followed by Tukey’s multiple comparison test. Different lower-case letters indicate significant difference at *P*<0.05.

To evaluate Cd accumulation, the transgenic lines were grown in a nutrient solution containing 0, 0.1, and 1 µM Cd for 8 d. The growth was almost similar between the transgenic and non-transgenic lines at either Cd concentration although B17 showed slightly less growth (Fig. 3A). The Cd concentration of the shoots was significantly decreased by 55% and 58%, respectively, at 0.1 and 1 µM Cd in the two transgenic lines compared with the non-transgenic line (Fig. 3B). By contrast, the Cd concentration in the roots of transgenic lines was significantly increased compared with the non-transgenic line at both Cd concentrations (Fig. 3C). The root-to-shoot translocation rate (shoot Cd content /total Cd content×100) of Cd was much lower in the transgenic lines than in the non-transgenic line (8% versus 20%) (Fig. 3D).

**Fig. 4.**
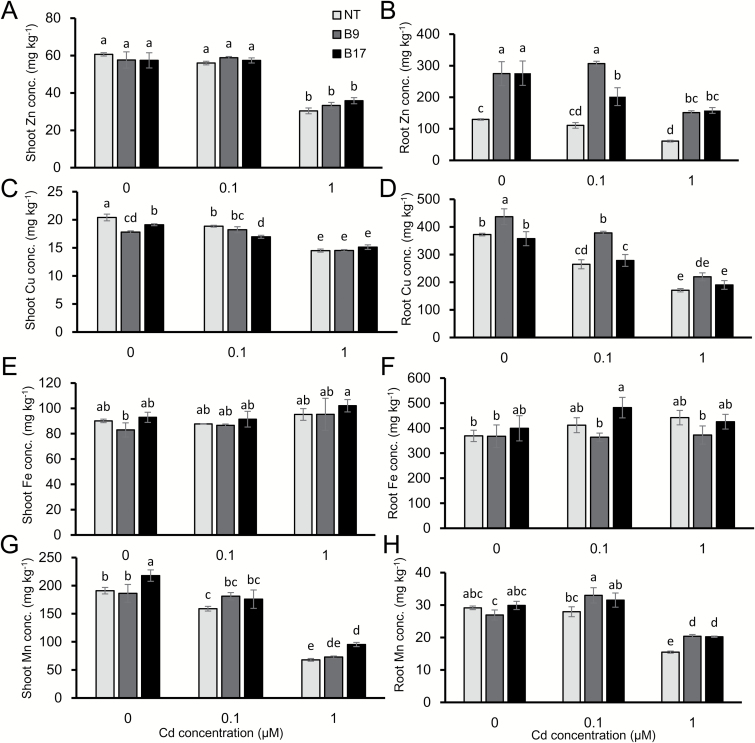
Concentration of essential metals in the roots and shoots. Seedlings (17 d old) of transgenic lines (B9, B17) carrying *OsHMA3* under the control of *OsHMA2* promoter were exposed to different Cd concentrations (0, 0.1, 1 µM) in a 1/2 Kimura B nutrient solution. After 8 d, samples were taken for determination of Zn (A, B), Fe (C, D), Cu (E, F), and Mn (G, H) in the shoots (A, C, E, G) and the roots (B, D, F, H) by ICP-MS. Data are means ±SD (*n=*3). Statistical comparison was performed by one-way ANOVA, followed by Tukey’s multiple comparison test. Different lower-case letters indicate significant difference at *P*<0.05.

There was no significant difference in the concentration of Zn, Cu, Fe, and Mn in the shoots between the transgenic and non-transgenic lines in both the absence and the presence of Cd (Fig. 4A, C, E, G). By contrast, the Zn concentration in the roots was higher in the transgenic lines than in the non-transgenic line (Fig. 4B) although the concentrations of Fe and Cu did not differ between the transgenic and non-transgenic lines at either Cd concentration. However, the Mn concentration in the roots of transgenic lines showed slight increase compared with the non-transgenic lines in the presence of Cd (Fig. 4D, F, H). This slight increase in Mn concentration of the roots of transgenic lines may be caused by unknown indirect effect. Overall, the concentration of Zn, Cu, and Mn was decreased by high Cd in both the roots and the shoots of the transgenic and non-transgenic lines (Fig. 4)

### Cd concentration in the xylem sap and root cell sap of transgenic lines

**Fig. 5.**
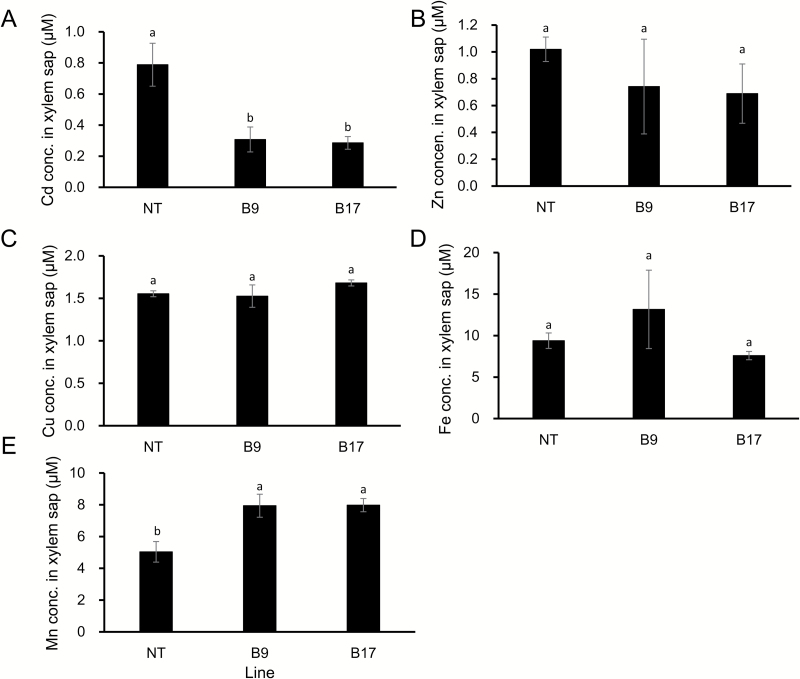
Concentration of metals in the xylem sap. Seedlings (17 d old) of transgenic lines carrying *OsHMA3* under the control of *OsHMA2* promoter were exposed to 1 µM Cd in a 1/2 Kimura B nutrient solution. After 8 d, xylem sap was collected with a micropipette. Concentration of Cd (A), Zn (B), Fe (C), Cu (D), and Mn (E) in the xylem sap was determined by ICP-MS. Data represent means ±SD (*n=*3). Statistical comparison was performed by one-way ANOVA, followed by Tukey’s multiple comparison test. Different lower-case letters indicate significant difference at *P*<0.05.

The Cd concentration in xylem sap was compared between the transgenic and non-transgenic lines. The concentration of Cd in the xylem sap of the transgenic lines was only two-fifths of the non-transgenic line (Fig. 5A). However, there was no significant difference in the concentration of Fe, Zn, and Cu between the transgenic and non-transgenic lines. The concentration of Mn was slightly higher in the xylem sap of the transgenic lines than that of the non-transgenic line (Fig. 5E), but the reason for this is unknown.

**Fig. 6.**
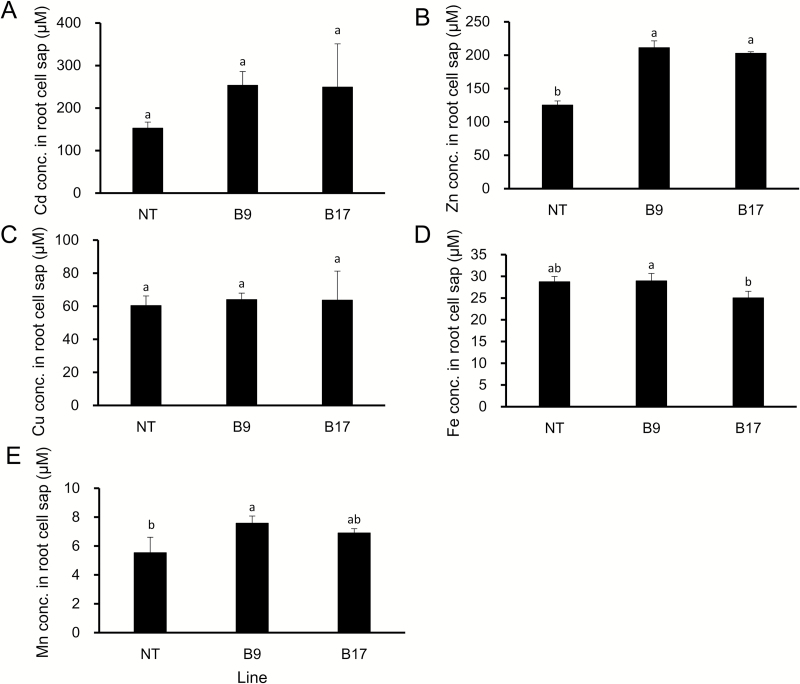
Concentration of metals in the root cell sap. Seedlings (17 d old) of transgenic lines carrying *OsHMA3* under the control of *OsHMA2* promoter were exposed to 1 µM Cd in a 1/2 Kimura B nutrient solution. After 8 d, the root cell sap was collected by centrifugation. Concentration of Cd (A), Zn (B), Fe (C), Cu (D), and Mn (E) in the root cell sap was determined by ICP-MS. Data represent means ±SD (*n=*3). Statistical comparison was performed by one-way ANOVA, followed by Tukey’s multiple comparison test. Different lower-case letters indicate significant difference at *P*<0.05.

Comparison of root cell sap showed that the Cd concentration in the root cell sap was 1.65-fold higher in the two transgenic lines than in the non-transgenic lines although the difference was not significant due to large variation (Fig. 6A). The concentration of Zn in the root cell sap was also higher in the two transgenic lines than in the non-transgenic line (Fig. 6B), but there was no difference in the concentration of Mn, Fe, and Cu between lines (Fig. 6C–E).

### Cd accumulation in straw and brown rice of transgenic lines

**Fig. 7.**
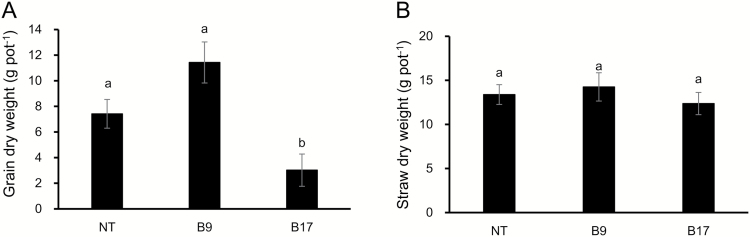
Growth and grain yield of transgenic lines. Both transgenic lines carrying *OsHMA3* under the control of *OsHMA2* and the non-transgenic line were cultivated in a Cd-contaminated soil till ripening. The grain yield (A) and straw (B) were recorded. Data represent means ±SD (*n=*3). Statistical comparison was performed by one-way ANOVA, followed by Tukey’s multiple comparison test. Different lower-case letters indicate significant difference at *P*<0.05.

**Fig. 8.**
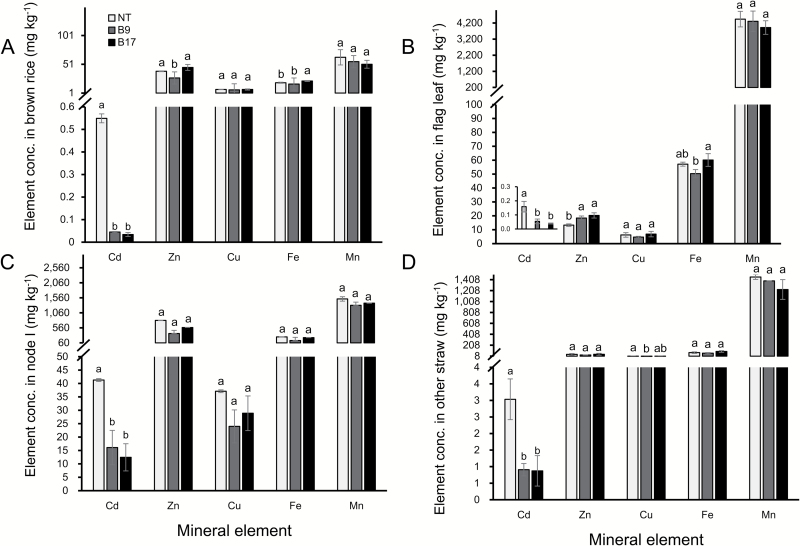
Concentration of Cd and other metals in different organs. Both transgenic lines carrying *OsHMA3* under the control of *OsHMA2* and the non-transgenic line were cultivated in a Cd-contaminated soil till ripening. The concentration of Cd, Zn, Cu, Fe, and Mn in the brown rice (A), flag leaf (B), node I (C), and remaining straw part (D) was determined by ICP-MS. Data represent means ±SD (*n=*3). Statistical comparison was performed by one-way ANOVA, followed by Tukey’s multiple comparison test. Different lower-case letters indicate significant difference at *P*<0.05.

To investigate the effect of the *OsHMA2* promoter on Cd accumulation in rice grain, both transgenic lines were cultivated in a Cd-contaminated soil and the Cd concentration was compared with the non-transgenic line. There was no significant difference in the grain yield between B9 and the non-transgenic line (Fig. 7A), but the grain yield of B17 was smaller compared with the non-transgenic line (Fig. 7A). There was no difference in the straw biomass between the transgenic and non-transgenic lines (Fig. 7B). The Cd concentration in brown rice of the non-transgenic line was 549 µg kg^−1^, whereas that in the transgenic lines was only 45 µg kg^−1^ in B9 and 34 µg kg^−1^ in B17 (Fig. 8A). The Cd concentration was also much lower in the node I, flag leaf, and remaining straw part of the transgenic lines compared with the non-transgenic line (Fig. 8B–D). However, there was no large difference in the concentration of Zn, Fe, Mn, and Cu between the transgenic lines and non-transgenic line in all organs (Fig. 8B–D).

### Expression of *ZIP* genes in the transgenic lines

Since Zn accumulation was increased in the roots but unchanged in the shoots of the transgenic lines compared with the non-transgenic lines, we investigated the expression level of six genes belonging to the ZIP family in the roots. Among them, the expression level of *OsZIP9* in the roots was significantly higher in the transgenic lines than in the non-transgenic lines (see Supplementary Fig. S3), whereas there was no significant difference in the expression of other *ZIP* genes between lines.

## Discussion

There are several ways to reduce Cd accumulation in rice grain, such as field remediation, water and nutrient management, and cultivation of low-Cd cultivars (Sebastian and Prasad, 2014). In the present study, we tested a novel transgenic approach for reducing Cd accumulation in the rice grain by expressing *OsHMA3* under the control of the *OsHMA2* promoter. *OsHMA3* encodes a tonoplast-localized transporter for Cd and Zn (Ueno *et al.*, 2010; Sasaki *et al.*, 2014). It is mainly expressed in the roots, but shows lower expression (Ueno *et al.*, 2010; Supplementary Fig. S2). Therefore, its capability for vacuolar sequestration of Cd in the roots is limited. On the other hand, *OsHMA2* is highly expressed not only in the roots, but also in the nodes (see Supplementary Fig. S2), and is one of the major translocation pathways of Zn and Cd in both roots and shoots (Yamaji *et al.*, 2013). Expression of *OsHMA3* under the control of the *OsHMA2* promoter resulted in significant increase of *OsHMA3* expression in the roots, shoots, and shoot basal region (including nodes) (Fig. 1A). This expression pattern is similar to native *OsHMA2* (Supplementary Fig. S2; Yamaji *et al.*, 2013). Furthermore, OsHMA3 localization in the transgenic line was altered; it was strongly localized at the root pericycle and phloem of enlarged and diffuse vascular bundles in the nodes (Fig. 2D–F). These results indicate that introduction of the *OsHMA2* promoter not only enhanced the expression level of *OsHMA3*, but also altered the tissue localization of OsHMA3. As a result, the Cd concentration in the grains of the transgenic lines was decreased to less than one-tenth that of the non-transgenic line when grown in a Cd-contaminated soil (Fig. 8A). The Cd concentration in the straw was also decreased to one-third of the non-transgenic line (Fig. 8D). Since rice straw is often fed to livestock, reduced Cd in the straw of transgenic lines will also contribute to limiting Cd transfer to the food chain.

The reduced Cd accumulation in the grain and straw could be mainly attributed to increased *OsHMA3* expression in the roots through sequestration of Cd into the vacuoles. This is supported by findings that the Cd in the root cell sap was increased (Fig. 6A) and that the Cd in the xylem sap was decreased (Fig. 5A). Localization of OsHMA3 in the nodes could also further reduce the accumulation of Cd to the shoots and grain. Recently, the node has been demonstrated to play a critical role in distribution of mineral elements including Cd in rice (Yamaji and Ma, 2014, 2017). Nodes have a complex, but well-organized vascular system, which consists of two major vascular bundles, enlarged vascular bundles and diffuse vascular bundles (Yamaji and Ma, 2014; 2017). To deliver mineral elements to the developing tissues such as young leaves and panicles, an inter-vascular transfer from enlarged vascular bundles to diffuse vascular bundles is required (Yamaji and Ma, 2014, 2017). Recently, a number of transporters involved in the inter-vascular transfer have been identified (Yamaji and Ma, 2017). Localization of OsHMA3 in the phloem of enlarged and diffuse vascular bundles of transgenic lines will help to sequester Cd into the vacuoles before reloading Cd from the intervening parenchyma tissues into the phloem of diffuse vascular bundles (Fig. 2F). This results in further efficient reduction of Cd in rice grain.

 Expression of *OsHMA3* under the control of the *OsHMA2* promoter did not significantly affect growth at the vegetative stage (Fig. 3A). The grain yield of the transgenic line B9 was comparable to the non-transgenic line although B17 showed a reduced grain yield (Fig. 7A). The exact reason for this negative effect is unknown, but one possibility is that other mutations occurred during transformation.

OsHMA3 is also involved in the sequestration Zn into the vacuoles of root cells (Sasaki *et al.*, 2014). We found that similar to Cd, the Zn concentration in the roots and root cell sap was higher in the transgenic lines than in the non-transgenic line (Figs. 4B, 6B). However, different from Cd, the Zn concentration in the shoots, xylem sap, and brown rice was similar between the transgenic and non-transgenic lines (Figs 4A, 5B, 8A). Unlike Cd, Zn is an essential element for plant growth and its homeostasis is tightly regulated (Krämer *et al.*, 2007). It was reported previously that overexpression of *OsHMA3* resulted in up-regulation of genes related to Zn uptake/translocation including *OsZIP4* (ZRT, IRT-like protein), *OsZIP5*, *OsZIP8*, *OsZIP9*, and *OsZIP10* (Sasaki *et al.*, 2014). In the present study, we found that *OsZIP9* is highly enhanced in the transgenic lines (see Supplementary Fig. S3). Although the exact role of *OsZIP9* is unknown, its up-regulation may contribute to maintaining Zn homeostasis in the shoots. The difference in up-regulation of *ZIP* genes between overexpression lines and *OsHMA2* promoter–*OsHMA3* transgenic lines may be attributed to different tissue specificity of *OsHMA3* expression. Different from *OsHMA2* promoter–*OsHMA3* transgenic lines (Fig. 2), *OsHMA3* was expressed in all tissues, which may cause more expression of *ZIP* genes to compensate Zn.

There was no difference in the concentration of Fe, Mn, and Cu in the shoots, roots, and brown rice between the transgenic and non-transgenic lines (Figs. 4, 8). However, high Cd in the external solution decreased the concentration of Mn and Cu in the shoots and roots of both transgenic and non-transgenic lines (Fig. 4C, G). Since Mn uptake is also mediated by OsNramp5 (Sasaki *et al.*, 2012), the decreased Mn accumulation is caused by competition between Mn and Cd for the OsNramp5. The mechanism for Cd-decreased Cu uptake remains to be further investigated since the transporter for Cu uptake in rice has not been identified.

 In conclusion, our results show that expression of *OsHMA3* under the control of the *OsHMA2* promoter enhanced the expression and altered the tissue localization of OsHMA3, resulting in efficient and significant reduction of Cd in rice grain through sequestering more Cd into vacuoles in the roots and nodes. This approach provides an effective way to selectively reduce Cd accumulation in the rice grain, which will ultimately contribute to reducing health risk.

## Supplementary data

Supplementary data are available at *JXB* online.

Fig. S1. Scheme of the construct used for the transformation.

Fig. S2. Expression pattern of *OsHMA2* and *OsHMA3* in different organs of rice.

Fig. S3. Expression of *OsZIP* genes in the roots of transgenic and non-transgenic lines.

Supplementary Figures

## Conflict of interest

All the authors have declared no conflict of interest.

## References

[CIT0001] Cheng F, Zhao N, Xu H, Li Y, Zhang W, Zhu Z, Chen M 2006 Cadmium and lead contamination in japonica rice grains and its variation among the different locations in southeast China. The Science of the Total Environment 359, 156–166. 16266740 10.1016/j.scitotenv.2005.05.005

[CIT0002] Clemens S, Ma JF 2016 Toxic heavy metal and metalloid accumulation in crop plants and foods. Annual Review of Plant Biology 67, 489–512. 10.1146/annurev-arplant-043015-112301 27128467

[CIT0003] Codex Alimentarius Commission of Food and Agriculture Organization 2006 Report of the twenty-ninth session of the Codex Alimentarius Commission. Rome: Codex Alimentarius Commission.

[CIT0004] Hiei Y, Ohta S, Komari T, Kumashiro T 1994 Efficient transformation of rice (*Oryza sativa* L.) mediated by *Agrobacterium* and sequence analysis of the boundaries of the T-DNA. The Plant Journal 6, 271–282. 7920717 10.1046/j.1365-313x.1994.6020271.x

[CIT0005] Ishikawa S, Ishimaru Y, Igura M et al 2012 Ion-beam irradiation, gene identification, and marker-assisted breeding in the development of low-cadmium rice. Proceedings of the National Academy Sciences, USA 109, 19166–19171. 10.1073/pnas.1211132109 PMC3511095 23132948

[CIT0006] Ishimaru Y, Takahashi R, Bashir K et al 2012 Characterizing the role of rice NRAMP5 in manganese, iron and cadmium transport. Scientific Reports 2, 286. 22368778 10.1038/srep00286 PMC3285952

[CIT0007] Krämer U, Talke IN, Hanikenne M 2007 Transition metal transport. FEBS Letters 581, 2263–2272. 17462635 10.1016/j.febslet.2007.04.010

[CIT0008] McLaughlin MJ, Parker DR, Clarke JM 1999 Metals and micronutrients food safety issues. Field Crops Research 60, 143–163.

[CIT0009] Miyadate H, Adachi S, Hiraizumi A et al 2011 OsHMA3, a P1B-type of ATPase affects root-to-shoot cadmium translocation in rice by mediating efflux into vacuoles. New Phytologist 189, 190–199. 20840506 10.1111/j.1469-8137.2010.03459.x

[CIT0010] Sasaki A, Yamaji N, Ma JF 2014 Overexpression of *OsHMA3* enhances Cd tolerance and expression of Zn transporter genes in rice. Journal of Experimental Botany 65, 6013–6021. 25151617 10.1093/jxb/eru340 PMC4203134

[CIT0011] Sasaki A, Yamaji N, Yokosho K, Ma JF 2012 Nramp5 is a major transporter responsible for manganese and cadmium uptake in rice. The Plant Cell 24, 2155–2167. 22589467 10.1105/tpc.112.096925 PMC3442593

[CIT0012] Satoh-Nagasawa N, Mori M, Nakazawa N, Kawamoto T, Nagato Y, Sakurai K, Takahashi H, Watanabe A, Akagi H 2012 Mutations in rice (*Oryza sativa*) heavy metal ATPase 2 (*OsHMA2*) restrict the translocation of zinc and cadmium. Plant & Cell Physiology 53, 213–224. 22123790 10.1093/pcp/pcr166

[CIT0013] Sebastian A, Prasad MNV 2014 Cadmium minimization in rice. A review. Agronomy for Sustainable Development 34, 155–173.

[CIT0014] Takahashi R, Ishimaru Y, Shimo H, Ogo Y, Senoura T, Nishizawa NK, Nakanishi H 2012 The OsHMA2 transporter is involved in root-to-shoot translocation of Zn and Cd in rice. Plant, Cell & Environment 35, 1948–1957. 10.1111/j.1365-3040.2012.02527.x 22548273

[CIT0015] Tang L, Mao B, Li Y et al 2017 Knockout of *OsNramp5* using the CRISPR/Cas9 system produces low Cd-accumulating indica rice without compromising yield. Scientific Reports 7, 14438. 29089547 10.1038/s41598-017-14832-9 PMC5663754

[CIT0016] Ueno D, Yamaji N, Kono I, Huang CF, Ando T, Yano M, Ma JF 2010 Gene limiting cadmium accumulation in rice. Proceedings of National Academy Sciences, USA 107, 16500–16505. 10.1073/pnas.1005396107 PMC2944702 20823253

[CIT0017] Uraguchi S, Kamiya T, Sakamoto T et al 2011 Low-affinity cation transporter (OsLCT1) regulates cadmium transport into rice grains. Proceedings of the National Academy Sciences, USA 108, 20959–20964. 10.1073/pnas.1116531109 PMC3248505 22160725

[CIT0018] Watanabe T, Shimbo S, Nakatsuka H, Koizumi A, Higashikawa K, Matsuda-Inoguchi N, Ikeda M 2004 Gender-related difference, geographical variation and time trend in dietary cadmium intake in Japan. The Science of the Total Environment 329, 17–27. 15262155 10.1016/j.scitotenv.2004.03.010

[CIT0019] Yamaji N, Ma JF 2007 Spatial distribution and temporal variation of the rice silicon transporter Lsi1. Plant Physiology 143, 1306–1313. 17259286 10.1104/pp.106.093005 PMC1820904

[CIT0020] Yamaji N, Ma JF 2014 The node, a hub for mineral nutrient distribution in graminaceous plants. Trends in Plant Science 19, 556–563. 24953837 10.1016/j.tplants.2014.05.007

[CIT0021] Yamaji N, Ma JF 2017 Node-controlled allocation of mineral elements in Poaceae. Current Opinion in Plant Biology 39, 18–24. 28558362 10.1016/j.pbi.2017.05.002

[CIT0022] Yamaji N, Xia J, Mitani-Ueno N, Yokosho K, Feng Ma J 2013 Preferential delivery of zinc to developing tissues in rice is mediated by P-type heavy metal ATPase OsHMA2. Plant Physiology 162, 927–939. 23575418 10.1104/pp.113.216564 PMC3668081

