# Supplementary material for: Effective reduction of cadmium accumulation in rice grain by expressing OsHMA3 under the control of the OsHMA2 promoter
Source: J Exp Bot. 2018 Mar 17;69(10):2743–52. doi: 10.1093/jxb/ery107 (PMC5920337; doi:10.1093/jxb/ery107)
Supplement: Supplementary Figures [file ery107_suppl_supplementary_figures_s1-s3.pdf]

Fig.S1

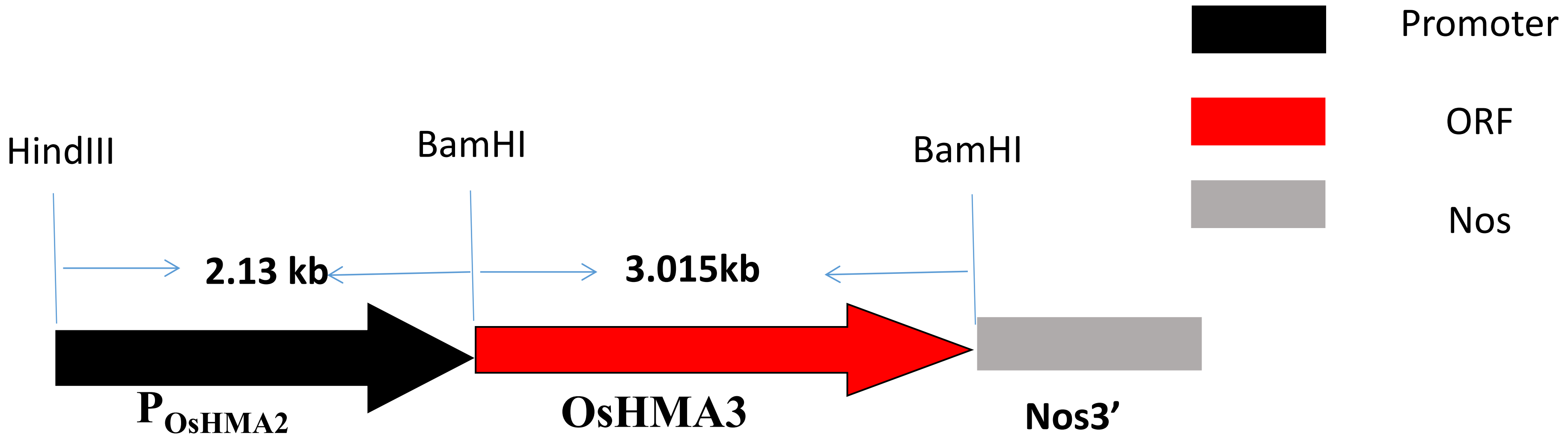

Supplementary Fig. 1. Scheme of the construct used for the transformation. *OsHMA2* promoter (2.13-kb) was fused with *OsHMA3* cDNA (3.015 kb).

Fig.S2

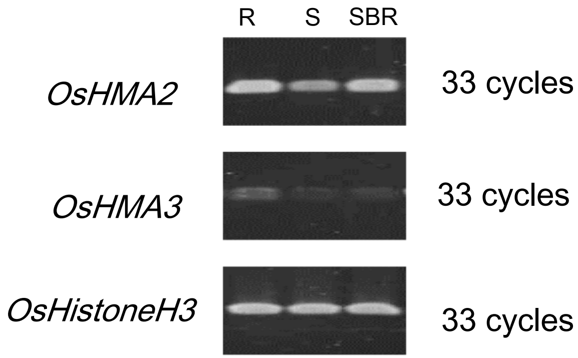

Supplementary Fig. 2. Expression pattern of *OsHMA2* and *OsHMA3* in different organs of rice. Samples of different organs including roots (R), shoots (S) and shoot basal region (SBR) were taken from seedlings (28-d-old, cv. Nipponbare). Semi-quantitative PCR was performed with 33 cycles. *HistoneH3* was used as an internal standard.

Fig.S3

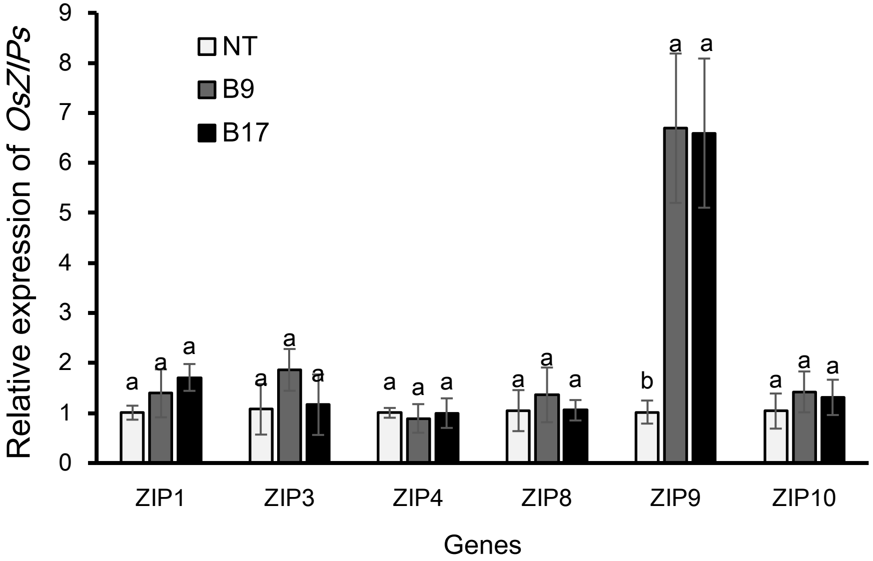

Supplementary Fig. 3. Expression of *OsZIP* genes in the roots of transgenic and non-transgenic lines. Root samples were taken from seedlings (28-d-old) of two independent transgenic lines carrying *OsHMA3* under the control of *OsHMA2* and non-transgenic (NT) line. The expression of six *ZIP* genes was determined by quantitative RT-PCR. *HistoneH3* was used as an internal standard. Expression relative to the non-transgenic line is shown. Data represent means±SD (n=3). Statistical comparison was performed by one-way ANOVA, followed by the Tukey's multiple comparison test. Different small letter indicates significant difference at $p < 0.05$.
